# Impact of COVID-19 on glaucoma management: A review

**DOI:** 10.3389/fopht.2022.1003653

**Published:** 2022-09-28

**Authors:** Manoj Yadav, Mukesh Tanwar

**Affiliations:** Department of Genetics, Maharshi Dayanand University, Rohtak, India

**Keywords:** glaucoma, telemedicine, teleglaucoma, vision, intraocular pressure

## Abstract

Glaucoma is the leading cause of irreversible vision loss and the second leading cause of blindness worldwide. The rapid transmission of SARS-CoV-2virus compelled governments to concentrate their efforts on emergency units to treat the large number of cases that arose due to the Covid-19 outbreak. As a result, many chronically ill patients were left without access to medical care. The progression of glaucoma in previously diagnosed cases has been accelerated; due to this, some have lost their vision. Evaluation of Covid-19’s effect on glaucoma treatment was one goal of this study. We used search phrases like “COVID-19,” “telemedicine,” and “glaucoma” to find published papers on COVID-19 and glaucoma. Artificial Intelligence (AI) may be the answer to the unanswered questions that arose due to this pandemic crisis. The benefits and drawbacks of AI in the context of teliglaucoma have been thoroughly examined. These AI-related ideas have been floating around for some time. We hope that Covid-19’s enormous revisions will provide them with the motivation to move forward and significantly improve services. Despite the devastation the pandemic has caused, we are hopeful that eye care services will be better prepared and better equipped to avoid the loss of sight due to glaucoma in future.

## Introduction

Glaucoma, the leading cause of irreversible blindness, is estimated to affect more than 60 million people worldwide, accounting for about 3 million cases of blindness. The optic neuropathy of glaucoma patients is characterized by relatively slow but progressive degeneration. Approximately fifty percent of patients are unaware of their disease (1, 2). Although elevated intraocular pressure (IOP) is the primary risk factor and the only modifiable risk factor for disease onset and progression, the pathogenesis of the disease is multifactorial and poorly understood (2, 3). However, not all patients with glaucoma have elevated IOP, and not all patients with elevated IOP develop glaucoma. Therefore, the pathogenesis of glaucoma is still not fully understood and likely differs among persons and populations. (2).

The researchers and clinicians focused on the pathophysiology and better treatment options for glaucoma, and the COVID-19 pandemic suddenly hit the world. Severe acute respiratory syndrome coronavirus 2 (SARS-CoV-2) is a highly contagious, single-stranded RNA virus that has rapidly spread across the globe. In severe cases, the resulting infection can cause a range of symptoms, including cough, fever, chest pain, and respiratory distress syndrome (4–6). Since its emergence in Wuhan, China, in late 2019, within months, on March 11, 2020, the World Health Organization (WHO) announced COVID-19 as a “pandemic” (7). With the non-linear rapid disease expansion, COVID-19 has caused widespread healthcare, socio-political and economic impact (8). As of July 20, 2022, there were 565,276,285 confirmed cases of COVID-19 worldwide, and 6,374,573 patients died of viral infection or other related complications (https://coronavirus.jhu.edu/).

## Early impact of COVID-19 outbreak on glaucoma care

A drastic escalation in the COVID-19 cases have increased the burden over health sector, due to which the majority of hospitals and clinics were converted into COVID-19 units to handle the emerging pandemic.

Aerosols, direct and indirect contact with respiratory droplets, virus in tears, and ocular secretions of infected individuals have been identified as the primary mode of transmission for SARS-CoV-2 (9, 10). Many medical societies worldwide have issued recommendations to discontinue routine diagnostic and surgical work to comply with government restrictions and reduce the risk of developing new cases. It was recommended that routine consultation and elective surgery be postponed, and only immediate or emergency care should be provided (11). Since ophthalmology is primarily concerned with elective surgery and ophthalmologists are among the medical specialties with the highest risk of COVID-19 infection, these changes have a significant impact on this medical specialty (9, 12–14). Ophthalmologists are at higher risk during the pandemic because they have to deal with patients which include physical contact during the examination. They are likely to catch the infection through the patients’ conjunctival and tear secretion. Studies have confirmed the strains of COVID virus in conjunctival and tear secretions of COVID-19-positive patients (9, 14, 15). Therefore, several national ophthalmology committees have recommended avoiding any non-urgent or non-emergency treatment to reduce person-to-person transmission of the virus (16).

As of 2010, 1 in 15 blind people was blind due to glaucoma highlighting the increasing global burden of glaucoma (17). The correct management of patients with glaucoma requires scheduled monitoring of IOP and visual field so that prompt intervention can be made in case of progression of damage (18). There is a reduction in 81% of medical visits during the first few months of the pandemic, including cataracts (-97%), sleep apnea (-91%) and osteoarthrosis (-88%), and glaucoma (-88%) (19, 20). As a result of the COVID-19 pandemic, ophthalmology departments from various countries have postponed 57% to 100% of glaucoma treatments, including Poalnd-100% (21), France-95% (4), Ireland-95% (22), Turkey-95% (23), Russia-97% (19), Portugal-90% (19), Spain-100% (24), and Italy-57% (16). Ophthalmology visits were reduced worldwide due to varying reasons, including fear of getting infected at the hospital, fear of using public transport in the pandemic, lack of swabs, delay in swab results, and unavailability of resources, staff, and supplies (11).

During the first phase of the pandemic, only patients who were at significant risk of irreversible visual morbidity on delaying surgery were operated. All patients got nasal swabs and serological tests to detect COVID-19 infection a week before the surgery. After surgery, chlorinated chemicals were used to disinfect the operating area, and a specific time gap was taken between cases to minimize any risk of virus infection (25).

## COVID-19 treatment and glaucoma progression

To reduce inflammation and prevent lung fibrosis, the majority of hospitalized COVID-19 patients were given systemic corticosteroids. Steroids have been linked to various long-term eye problems, such as cataracts and glaucoma (26–28). The linked risk seems to increase with increasing dose and duration (29). Corticosteroids like dexamethasone and prednisolone, commonly used in COVID-19 patients, have a higher propensity to produce glaucoma and cataracts. (30). Glucocorticoids can disrupt the trabecular meshwork and decrease the outflow facility, resulting in elevated intraocular pressure (31). Genetic susceptibility plays a crucial role in this aspect, as certain individuals are more sensitive to steroid-induced problems. Therefore, the use of systemic steroids due to COVID-19 may increase the incidence of glaucoma cases requiring treatment, resulting in an increased demand for antiglaucoma drugs and glaucoma surgery (32).

## Visual field artifacts due to use of face mask

Patients and employees were advised to wear masks that cover their noses and mouths to limit the chance of respiratory droplet infections (33). The improper way of using face masks became a new cause of visual artifacts, and this problem increased with the increasing use of face covers due to the elevation in COVID-19 cases worldwide (33–35). Improper use of face masks can interfere with the ophthalmic testing results. An inferior visual field defect can occur due to the edge of the mask riding up the patient’s face, and in some cases, defects occur due to fogging of the trial lens of the perimeter (33, 35). These mask-related artifacts can mimic the pathological defects seen in glaucomatous patients, which may lead to an unnecessary referral or additional testing, follow-up visits, and advancement of therapies related to glaucoma, which is not advisable during the pandemic (33).

Another mask-related problem is discomfort and difficulty in breathing, which hinders accurate testing of visual field defects (34). To deal with these mask-related artifacts, patients were advised to use tape on the bridge of the nose (34, 36), use of double masks (37), or wear a surgical mask (38). To allow the exhaled air to escape away from the patient, it was advised to knot the superior tie below the ears and the inferior one above the ears which will create two lateral vents for the exhaled air to escape and minimize the chances of fogging on the lens, but this removes most of the benefit of wearing a mask, especially regarding transmitting the virus (39). Using a closed chamber perimeter to evaluate glaucoma patients with intolerance to masks during visual fields execution is the best way to deal with mask-related artifacts until the pandemic is over (35).

## Impact of COVID-19 on glaucoma diagnosis

The COVID-19 pandemic has a remarkable effect on the global healthcare system, restricting the continuous care of patients with chronic diseases, affecting patients’ health-seeking behavior, and influencing surgeons’ assessment skills (40). Various parameters have been established to lessen the probability of contamination. Cross-contamination can be avoided by utilizing tonometers with disposable tips (41). Noncontact tonometers should be avoided because they generate micro aerosols that can disperse the virus and increase the probability of contamination (42). As direct ophthalmoscopes are in close proximity to the patient’s face and mouth, indirect ophthalmoscopy with a 20D lens should be utilised in COVID-19-positive cases. If possible, tab-based or virtual reality perimetry can be used instead of traditional perimetry. For suspects and glaucoma patients, imaging is preferable to visual fields because there is a lower risk of cross-contamination, sanitization is simpler, and the test is quicker (43).

As some studies indicate the possibility of virus in the conjunctival sac secretion of COVID-19 patients, the importance of disinfecting instruments is emphasised (44, 45). Instruments, patient and technician interface surfaces are to be disinfected using 0.5% bleach, sodium hypochlorite (for tonometer) (46) or 3% hydrogen peroxide, ethylene oxide (in the case of lenses), and 70% isopropyl alcohol to wipe eyepatch, chinrest, headrest, trial lens holder, trial lens, and patient response button after perimetry (43, 47).

## Impact of COVID-19 on glaucoma treatment, follow-up and medication

The pandemic has a significant psychosocial impact on the general population, magnified in patients with chronic disease. Chronic disease patients’ stress and anxiety levels have increased due to their self-isolation, movement restrictions, and fear of contracting an infection, which impacts their disease progression and medication adherence (48–50).

Non-adherence to treatment was prevalent among patients with POAG during the COVID-19 pandemic in various studies conducted on the different populations, including India (50), Croatia (51), United States (52), Egypt (53), and Pakistan (54). Before the pandemic, the main barriers to glaucoma medication adherence were cost, difficulty with drop instillation, and forgetfulness. However, as the pandemic struck and strict lockdowns were implemented, the percentage of patients who adhered to their glaucoma medications changed. Lack of availability or accessibility of medication (50, 55–57), travel restrictions related to the pandemic (50), financial difficulties (enormous economic burden of the pandemic) (50, 53), and a lack of knowledge and understanding of glaucoma were the primary causes of this drastic change (50, 52, 53).

Regular and thorough monitoring is essential for this potentially blinding condition. The new WHO regulations to combat COVID-19 had a significant impact on the follow-up appointments of glaucoma patients (53, 58). The scheduled postoperative follow-up visits were decreased by 43.9% (59). The barriers to a regular follow-up during the pandemic include fear of contamination during follow-up visits (53), lockdown with transportation restrictions, financial difficulties (50, 54), unavailability of medical staff and clinics as most of the hospitals were converted into COVID centers (40, 50, 60).

## COVID-19 and glaucoma severity

Glaucoma needs timely diagnosis and treatment to eliminate the chances of visual field loss. The importance of routine follow-up in the therapy of glaucoma has been well established by numerous studies, which show a correlation between glaucoma severity worsening and inadequate follow-up (61, 62). In a developing nation like India where glaucoma follow-up is notoriously poor, the pandemic has made the condition even worse and placed a tremendous burden on the ability to provide glaucoma care continuously (50). Due to the imposed restrictions in pandemic, only emergency treatment facilities were available to reduce person-to-person transmission of the virus (16). In a study conducted in South India, the number of high-risk patients and new cases presenting as emergencies increased by 48.9% and 48.4%, respectively, compared to 2019. Patients presented with more severe vision loss, higher intraocular pressure (IOP), advanced cataracts, and significant optic disc injury compared to the prior year, suggesting that the severity of eye emergencies had increased. Patients had worse mean uncorrected VA (logMar 1.6 ± 1.1 vs. 1.4 ± 1.0; P < 0.001) and higher mean IOP (26.9 ± 15.9 mm Hg vs. 23.0 ± 13.3 mm Hg; *P* < 0.001) during the lockdown period compared to the previous year (59) and similar results were found in a Turkish study where the mean IOP increased from 29.94 ± 10.49 mm Hg to 32.9 ± 13.3 mm Hg during the lockdown period (63). In another study from India, the severity of glaucoma due to the pandemic was evaluated using automated perimetry visual field index assessments, which revealed that 78.13% of patients had experienced significant visual field loss, 81.25% of patients had elevated IOP, and 59% of patients had increased cup disc ratios (Bala* and Islam).

## Glaucoma surgery in COVID-19 era

The majority of ophthalmologists and patients face the dilemmas of avoiding or minimizing irreversible visual loss due to the progression of diseases and delayed treatment while recognizing that hospital visits increase the risk of viral transmission for patients and close contacts (40, 50, 64, 65). Numerous glaucoma surgical interventions have been halted in mild-to-moderate cases with no evidence of progression or immediate visual threat. Restrictive measures and concerns about the risk of exposure for medical personnel had a substantial impact on the selection of anesthesia and surgery (40). Less invasive glaucoma procedures requiring fewer postoperative visits and fewer postsurgical interventions were favoured at the discretion of the operating surgeon (64, 65).

Due to COVID-19, fewer glaucoma-related surgical procedures have been performed (66). The medical records of patients scheduled for glaucoma surgery were evaluated by specialized medical personnel. Those at risk for rapid glaucoma progression, angle-closure glaucoma, and those with a significant rise in intraocular pressure were identified and deemed surgical candidates (66). The time of surgery, less overall patient contact to reduce the risk of COVID-19 transmission, less postoperative follow-up, and fewer postsurgical interventions were the factors behind the modification of surgical practices after the onset of COVID-19 (40, 65, 66).

Prior to COVID-19, trabeculectomy was the most favored technique for glaucoma (67), with a varying percentage of glaucoma specialists from various countries like 87% in the United Kingdom (66) and 62.8% in Italy (65). During the pandemic, however, fewer trabeculectomies, glaucoma drainage devices, and minimally invasive glaucoma surgeries were performed. Micropulse and traditional transscleral cyclodiode were the most frequent alternatives (59, 66). A study conducted in the United Kingdom, determined that the adoption of micropulse diode was encouraged by the reduced postoperative follow-up (according to 90% of glaucoma specialists) and shorter surgical time (66). The number of minimally invasive glaucoma surgeries (MIGS), comprising implantation of the Preserflo Micro Shunt, increased from 8.5% before COVID-19 to 22.5% after COVID-19 (65). In an effort to save time and prevent a potentially aerosol-generating treatment, the frequency of combined phacoemulsification and antiglaucoma procedures declined, whereas the frequency of glaucoma surgery alone increased (59, 66).

## Teleglaucoma in COVID-19 era

Teleglaucoma refers to the use of telemedicine to detect and manage people with glaucoma or at risk for the disease. In 1999, the first study about teleglaucoma was published (68, 69). Telemedicine may be synchronous (real-time video conferencing between the provider and patient), asynchronous (data captured and then transmitted to the physician for remote assessment), or a hybrid of the two (70). Teleglaucoma can assist in screening (71), diagnostic consultation (72, 73), and long-term treatment monitoring (74–76). The most typical measurements involved in teleglaucoma include fundus images (76–79), automated visual field testing (72, 76, 78), IOP (72, 76, 79), visual acuity (76, 79, 80), Slit lamp examination (76, 77), corneal thickness (1, 72, 78), and Optical coherence tomography  (77, 78)

Telemedicine has taken center stage due to the ongoing COVID-19 pandemic, which has altered the pattern of healthcare delivery. Due to the ongoing epidemic, routine outpatient services and elective procedures have been discontinued, and only emergency inpatient care is being offered (1, 80). Tele ophthalmology, which had been a bystander for more than a decade, became a necessity. Not only does it reduce the need for the patient and clinician to be in the exact location, but it also protects both the patient and the doctor by decreasing the doctor’s patient exposure time and restricting the patient’s hospital visits to just grave emergencies (71, 78, 79). It has served as a lifeline for about two months, during which the ordinary services were completely halted.

Convenience, shorter travel time to medical clinics (74), better access to specialized care for glaucoma (81), and decreased patient costs (71, 77, 78, 82, 83) are among the benefits cited in the literature. The benefits are primarily observed in underserved or isolated regions, rural or remote locations where there are few ophthalmologists (76, 78). It is difficult to exclude the potential educational applications of teleglaucoma as a separate aspect. The vast number of images obtained using a teleglaucoma model can easily be used to broaden the education of medical students, residents, and fellows. Incorporating telemedicine into medical education has produced favorable benefits (84, 85). Teleglaucoma screening with optic nerve examinations has been shown to be highly sensitive (95 percent confidence interval [CI]: 0.77, 0.88] and specific (95 percent CI): 0.79), according to research done by Thomas and colleagues  (71, 86). It has also been proven that teleglaucoma accurately diagnoses 83.3 percent of the cases of glaucoma and correctly classifies 79 percent of the people who do not have the disease as glaucoma-free (71).

## Challenges and limitations of teleglaucoma

Teleglaucoma is the need of the hour, and despite its advantages, it also has several downsides and limitations. As the application of AI in medicine is in its infancy, patients’ and physicians’ faith in new technologies is crucial (87, 88). Because errors and problems in health care receive more media coverage than accomplishments, clinicians are hesitant to utilize telemedicine more prominently. The main limitations of teleglaucoma are a lack of budget and infrastructure (71, 89), data privacy, and liability (86, 90, 91), digital exclusion of low household income and older age people (92), lack of digital knowledge in patients as well as clinicians, shortage of telecommunication devices, specificity and consistency (86). The use of teleglaucoma is significantly more problematic in emerging and underdeveloped nations, where the majority of rural residents lack basic necessities like reliable energy, internet access, and smart telecommunication gadgets (93). Studies have indicated that ophthalmologists had low confidence since the information presented during the teleconsultation was highly variable compared to the in-person consultation (72, 93). So, all these above-mentioned points are a barrier to the broad range use of AI in the health sector.

## Discussion

One of the major causes of permanent blindness worldwide, glaucoma, can be exacerbated by delays in diagnosis and treatment. As the COVID-19 pandemic struck, the health sector shifted its focus to finding a cure of the viral infection. Many problems in the ophthalmology field arose as a result of this pandemic. Only the emergency medical facilities were available due to the strict lockdowns, and the number of new glaucoma consultations and follow-ups dropped dramatically. Patients’ adherence to glaucoma medication and follow-up has declined worldwide due to the pandemic’s social and economic impact. All these issues can be managed up to some extent in such pandemic times by normalizing the use of artificial intelligence in the medical sector. AI-enhanced telemedicine could lead to a new era of safe, tailored, efficient, and cost-effective care. In high-risk populations and areas with limited resources, teleglaucoma screening programmes can be implemented in a variety of ways to complement existing therapeutic approaches. Glaucoma management costs a lot of money in most countries because of the high cost of professional ophthalmologists. The glaucoma specialists could spend more time on tough cases if technology could take over part of the tiresome tasks they do when caring for suspicions or patients with early glaucoma. Teleglaucoma may be helpful in remote places where glaucoma specialists are not readily available for face-to-face treatment. Teleglaucoma’s importance cannot be overstated, despite its drawbacks and limits. Continuous advances in computing power and information technology may be able to help address some of our current issues. Prior to widespread use, teleglaucoma screening and management need to improve its sensitivity and specificity. Teleglaucoma can be used to monitor glaucoma suspects and to keep track of individuals with moderate and stable glaucoma or those who need frequent monitoring in pandemic situations. Although technological improvements have resolved most of our questions regarding the usage of teleglaucoma, there is still work to be done before it replaces the traditional methods of patient monitoring.

## Author contributions

The first author has gathered the related articles and done the writing work. The corresponding author has guided throughout the manuscript and revised the manuscript. All authors contributed to the article and approved the submitted version.

## Funding

Senior Research Fellowhip, Council of Scientific and Industrial research, New Delhi, India. Grant no: 09/382(0243)/2019-EMR-I.

## Conflict of interest

The authors declare that the research was conducted in the absence of any commercial or financial relationships that could be construed as a potential conflict of interest.

## Publisher’s note

All claims expressed in this article are solely those of the authors and do not necessarily represent those of their affiliated organizations, or those of the publisher, the editors and the reviewers. Any product that may be evaluated in this article, or claim that may be made by its manufacturer, is not guaranteed or endorsed by the publisher.
